# The Importance of Assessing Nutritional Status to Ensure Optimal Recovery during the Chronic Phase of Stroke

**DOI:** 10.1155/2018/1297846

**Published:** 2018-01-11

**Authors:** Monica C. Serra

**Affiliations:** Atlanta VA Medical Center and Emory University School of Medicine, Atlanta, GA, USA

## Abstract

**Background:**

Despite evidence that many of the consequences of stroke that hinder recovery (i.e., obesity, muscle atrophy, and functional declines) have nutritionally modifiable behavior components, little attention has been focused on the significance of nutrition beyond the acute phase of stroke.

**Objective:**

This literature review summarizes the evidence for and against the influence of nutrition on optimal recovery and rehabilitation in chronic (>6 months) stroke.

**Results:**

The literature, which is mainly limited to cross-sectional studies, suggests that a suboptimal nutritional status, including an excess caloric intake, reduced protein intake, and micronutrient deficiencies, particularly the B-vitamins, vitamin D, and omega 3 fatty acids, may have deleterious effects on metabolic, physical, and psychological functioning in chronic stroke survivors.

**Conclusions:**

Careful evaluation of dietary intake, especially among those with eating disabilities and preexisting malnutrition, may aid in the identification of individuals at increased nutritional risk through which early intervention may benefit recovery and rehabilitation and prevent further complications after stroke.

## 1. Introduction 

Since stroke is the leading cause of long-term disability [1], identifying and managing potential risk factors that may hinder a full recovery remain to be of great importance. Although literature is available supporting a role of inadequate nutrition in elevated stroke risk [2] and poor stroke recovery during the acute-stage [3], little attention has been focused on the significance of suboptimal nutrition on the development of metabolic, physical, and psychological dysfunction in chronic (>6 months) stroke. This is in spite of evidence that many of the consequences of stroke that hinder recovery (i.e., obesity, muscle atrophy, and functional declines) have nutritionally modifiable behavior components. Chronic stroke survivors are at greater risk for developing nutrition-related chronic disease, including Type 2 Diabetes Mellitus (T2DM) and osteoporosis [4, 5]. Further, reduced physical and cognitive functioning and depression, which are more common among stroke survivors than healthy age-matched adults, are influenced by nutritional status [6, 7]. Thus, promoting optimal nutrition can enhance the efficacy of stroke rehabilitation through their positive influence on metabolic, physical, and mental functioning.

This literature review focuses on the assessment of nutritional status, factors that affect nutritional risk, and the possible consequences of poor dietary patterns on optimal recovery and rehabilitation in chronic stroke. Of importance to note is that ischemic stroke comprises 80–85% of all stroke cases and only 15–20% of the cases are hemorrhagic [8]; thus, the majority of the available literature is limited to ischemic stroke.

## 2. Assessment of Nutritional Status in Chronic Stroke

Discrepancies in how nutritional risk is defined (i.e., variations in inclusion of subjective reporting of dietary intake and objective anthropometric measurements and laboratory tests) are known to affect the categorization of malnutrition following acute stroke [9] and are likely similarly relevant in chronic stroke. Traditional methods for assessing dietary intake (i.e., food records, food frequency questionnaires (FFQs), and 24-hour recalls) rely on information reported directly by a patient. However, the accuracy of these methods may be diminished by numerous factors after stroke, including cognitive and visual impairments and apraxia [10] and reliance on caregiver reporting. Further, medications and comorbidities may affect the absorption and utilization of nutrients. Thus, assessment of more objective measures of nutritional status also may be useful.

Objective measures, including anthropometric (i.e., body weight, BMI, skinfold, or arm circumference) and biochemical clinical indictors (i.e., albumin, prealbumin, transferrin, and lipids) also may be monitored at baseline and as changes occur over time to assess nutritional status. Change in body weight is the most common anthropometric measure of malnutrition used, with weight loss associated with low albumin and prealbumin values after stroke [11]. It is observed that ~45% of stroke survivors lose > 3 kg of body weight within the first 16 months after their stroke onset [11]. Albumin is the most common biomarker used to assess malnutrition, with albumin concentrations found to be lower in chronic stroke survivors than in age-matched nonstroke controls [12]. Clinicians should be aware of the strengths and limitations of each measure and should likely use a combination of markers instead of a single one to assess nutritional risk.

To overcome problems of definition, numerous screening assessment tools are available. The subjective global assessment (SGA) [13] and mini nutritional assessment (MNA) [14] are the most frequently used nutritional screening tools used in hospitalized stroke patients [15]. Although both are validated for use in disease or injury states, neither is validated specifically in a stroke population [9]. It appears that the MNA predicts a higher prevalence of nutritional risk and may more accurately predict length of stay in an older geriatric population; however, it may not be appropriate in those who cannot provide reliable self-assessment such as those with confusion or serious poststroke aphasia and apraxia [16]. Using screening assessment tools, there is evidence that 12–41% of stroke survivors are at risk for malnutrition at six months [17–19] and 11% at 16–18 months [11, 20], with limited data beyond the first two years after stroke. The following sections will outline the justification for the need to better understand nutritional risk in chronic stroke survivors as poor nutrition can have negative consequences on stroke recovery.

## 3. Factors Affecting Dietary Intake and Nutritional Risk during Chronic Stroke Recovery (Figure 1)

Upon entering the chronic phase of stroke, the prevalence of preexisting medical conditions, such as malnutrition, obesity, and comorbidities, may affect a survivor's current nutritional status by altering nutritional needs. Underweight and malnourished stroke patients are more likely to be dependent, have medical complications, and have lower functional (i.e., self-care and mobility) outcomes at six months [3, 21, 22]. Conversely, those that are obese and with other chronic conditions are less likely to demonstrate improvements in motor impairment and functional mobility performance [23]. These data indicate that those at either extreme of the BMI spectrum entering into the chronic phase of stroke are at elevated nutritional risk.

A greater severity of stroke can continue to compound baseline nutritional inadequacies by affecting an ability to eat due to physical limitations. Eating problems (i.e., problems with chewing, swallowing, and leakage of food from the mouth) and loss of upper arm function contribute greatly to reduced caloric intake. Other physical factors that could affect the ability to carry out activities required to eat a meal after stroke include impairments with postural control, vision, and cognition. Studies suggest that in the chronic phase of severe stroke ~30–50% of survivors require assistance with eating and 5–10% require to be fed [24].

A stroke also can alter the desire to eat. Anxiety and depression, which may occur with the inability to return to prestroke independence levels [28], are associated with weight loss [29] and lower appetite scores [17] in stroke survivors. Further, the initiation or combination of several medications (stroke survivors are discharged home on an average of 11.3 medications [30]) is associated with lack of appetite, xerostomia, and the potential for constipation, which may affect the desire to eat. This impedance to dietary intake may predispose stroke survivors to a suboptimal nutritional status upon entering the chronic phase of recovery.

## 4. Consequences of Inadequate Nutrition in Chronic Stroke

Since the majority of nutritional risk factors present for primary stroke are the same for secondary stroke prevention, including diets rich in fruits and vegetables (antioxidants and dietary fiber), fish and milk, B-vitamins, potassium, calcium, and magnesium and lower in sodium and dietary fat, and are available elsewhere [2], this review will focus on the importance of assessing nutritional status to prevent metabolic, physical, and psychological dysfunction necessary for optimal rehabilitation and recovery in chronic stroke (summary provided in Table 1).

### 4.1. Nutritional Status and Metabolic Dysfunction

#### 4.1.1. Sarcopenic Obesity

Chronic hemiparesis is present in nearly 50% of all long-term ischemic stroke survivors [31]. Hemiparesis may result in body composition changes, including increases in fat mass and decreases in fat-free mass in the paretic versus the nonaffected leg [32]. While not always observed [33], we [34] and others [35] show that resting metabolic rate (RMR), the main contributing component of total daily energy expenditure, is ~10–20% lower than predicted and is related to fat-free mass in chronic stroke survivors with hemiparesis, indicating that muscle atrophy after stroke may lead to a reduced RMR.

A reduced RMR, as well as a sedentary lifestyle [36], may predispose stroke survivors to obesity by reducing daily caloric requirements. Indeed, by one year after stroke, the prevalence of obesity may be as high as ~40% [37]. Obese stroke survivors are more likely to have elevated vascular risk factors, including T2DM, depression, hypertension, low HDL cholesterol, and obstructive sleep apnea [4]. However, it must be recognized that functional recovery [38, 39] and 10-year survival [40] are significantly better among those that are overweight and obese than those who are of normal weight, highlighting an obesity paradox with regard to body weight and recovery in stroke.

Sarcopenic obesity is related to reduced functional performance, elevated inflammatory profiles, a worse psychological status, and a reduced quality of life [41]. Ensuring an optimal nutritional intake of calories and protein is essential to maintain muscle mass and preventing the development of obesity and other chronic diseases. There is evidence that, at six months after stroke, survivors (44% were living in institutions, 17% were supported in their own homes by nonresidential carers, and 39% had live-in care support) are consuming 81% and 163% of the Estimated Average Requirement (EAR: the daily intake that is estimated to meet the nutrient requirement of half the healthy individuals in a life stage and sex group) for energy and protein, respectively [6]. While the protein intake appears adequate from a quantitative standpoint, the biological value (i.e., measurement of protein quality) of the protein, which is known to greatly influence physiological function in the body, is unknown and appears of importance to future investigation.

#### 4.1.2. Fracture

The incidence of hip fracture after a stroke is reported to be at least 1.5 times higher (evidence of up to 7 times higher) than in the general population [42–44]. Hip fractures in stroke survivors are associated with poorer outcomes, including a 3-fold higher incidence of postoperative myocardial injury and the need for institutionalization compared to nonstroke controls [45]. While physical inactivity plays a large role in bone density, formation, and strength, several nutrients also are indicated. Protein, calcium, vitamin D, phosphorus, magnesium, vitamin K, zinc, and the B-vitamins are associated with the formation, construction, and maintenance of healthy bones. Although the relationship of the majority of these nutrients to bone density and/or fracture risk specific to stroke risk is unknown, several are implicated in the elevated fracture risk.

A high prevalence of vitamin D insufficiency and excess bone resorption is observed in stroke survivors [45–47]. Because of impaired mobility, especially outdoors, sunlight exposure is often reduced, resulting in a subsequent vitamin D deficiency [48]. However, even when normal vitamin D levels are observed, significant bone mineral density loss may occur after stroke [49]. Further, the rates of osteoporosis are similar between stroke survivors with and without vitamin D deficiency [50], suggesting that vitamin D status is not the only factor affecting bone metabolism. In vitamin D-deficient older stroke patients, a low vitamin K status may further increase fracture risk, especially on the paretic side [51].

Elevated homocysteine levels, which are observed in a third of stroke survivors [52], also are implicated in elevated fracture risk [53]. Lower intakes of the B-vitamins (folate, vitamin B6, and vitamin B12) are associated with higher homocysteine levels. Approximately 25% of stroke survivors have low plasma B-vitamin status [54], indicating that stroke survivors may be at risk for fracture due to low B-vitamin status. Additionally, alcohol use may destroy B-vitamins, with ~15% of stroke survivors drinking more than the weekly recommendation for alcohol [37]. Conversely, higher serum vitamin B12 levels (>475 pg/mL) also are related to several adverse events, including greater risk of hip fracture risk among stroke survivors [45], but the mechanism for this elevated risk is not currently understood.

These data suggest that the impact of specific nutrients on bone health is not well elucidated in stroke survivors. However, they indicate that early monitoring of the plasma concentrations of vitamins D and K and the B-vitamins may aid in the identification of elevated fracture risk following a stroke.

### 4.2. Nutrition Status and Physical Dysfunction

In survivors six months after discharge from a stroke, a suboptimal nutritional status is associated with having difficulty buying and ingesting food and fatigue [18]; and decreased caloric and protein intakes are associated with physical impairment and disability [6]. Further, stroke survivors with eating problems are more likely to have dependency in other activities of daily living [55]. In addition to loss of skeletal muscle mass, inadequate caloric intake and poor diet quality can lead to weakening of respiratory muscles and compensatory declines in cardiac function, which may affect the ability for patients to participate in and benefit from rehabilitation.

Although the consumption of fish and/or fish oils, specifically omega-3 long chain polyunsaturated fatty acids, by chronic stroke survivors is not documented, nonstroke populations suggest that higher plasma omega 3 concentrations protect against accelerated age- and disease-associated decline of physical performance and muscle mass [56, 57]. There is some suggestion that the mechanism of action of omega 3s on physical function and muscle composition may be through its effects on inflammation and oxidative stress [58]. The associations of other antioxidants and anti-inflammatory nutrient status (i.e., vitamins C and E) in nonstroke populations on the prevention of muscle loss appear equivocal and are reviewed elsewhere [59, 60]. Currently, there is insufficient evidence supporting the assessment of omega 3 or other anti-inflammatory or antioxidant nutrient profiles as a marker of poor physical functioning after stroke.

### 4.3. Nutritional Status and Psychological Dysfunction

#### 4.3.1. Depressive Symptoms

Depression is prevalent in ~25–30% of chronic stroke survivors [61]. In older (>70 years old) survivors ~2 years after stroke, survivors with elevated homocysteine concentrations are twice as likely to be classified as clinically depressed [52]. Low folate and vitamin B12 deficiency are implicated in this process as both are required for the biosynthesis of serotonin, dopamine, and norepinephrine, as well as in cerebrovascular burden [24]. Dopamine is made from the amino acid tyrosine and serotonin from tryptophan; thus if there is a lack of any of these two amino acids, there will not be enough synthesis of the respective neurotransmitters identifying the need for adequate protein. Additionally, significant correlations between lower fish consumption and higher rates of depression are observed in nonstroke populations [62]. Omega 3s plays a role in modulating synaptic membrane receptor function in the central nervous system, especially in the cerebral cortex [63]. This alteration can disrupt basic neurotransmitter activity, including serotonin. Finally, there is evidence that at six months after stroke, those with the lowest vitamin D concentrations may have a higher prevalence of depressive symptoms [64]. The mechanism whereby vitamin D may be associated with depression is not clearly understood. There are vitamin D receptors in the hypothalamus, which may be important in neuroendocrine functioning [65]. Several studies identify an unfavorable influence of depression on functional outcomes after stroke [66, 67], indicating the potential benefit of assessing and addressing nutritional inadequacies associated with depression in stroke survivors undergoing rehabilitation focused on functional recovery.

#### 4.3.2. Cognitive Impairment

Approximately 16% of chronic stroke survivors are cognitively impaired [68]. It is observed that the community ambulation rate is higher in cognitively normal stroke survivors at six months after stroke than those that have a cognitive deficit [69]. Further, while it is determined that rehabilitative care is equivalent for patients with and without cognitive impairment at six months, those that are cognitively impaired stroke patients experience worse recovery of activities of daily living [7].

High plasma homocysteine concentrations are an independent risk factor for cognitive impairment and dementia [70, 71]. There are many possible mechanisms by which homocysteine may affect cognition, including effects on cerebral vasculature and inflammation [72]. Lower B-vitamin intake is associated with greater cognitive dysfunction in older adults, some of which had a history of stroke [73]. Similar results are observed between low fish intake and greater cognitive dysfunction in acute stroke survivors [74], but this relationship is currently unexplored in chronic stroke. Possible mechanisms include the role of omega 3s on neuronal functioning, inflammation, and oxidation [75]. Further, in nonstroke populations, there is evidence that deficiencies in antioxidants such as vitamins C, D, and E and beta-carotene, as well as the presence of nutrition-related disorders such as hyperlipidemia, hypertension, and T2DM could be some of the nutrition-related risk factors for cognitive impairment [76].

## 5. Conclusion

These data indicate that a suboptimal nutritional status, including an excess caloric intake, reduced protein intake, and micronutrient deficiencies (particularly the B-vitamins, vitamin D, and omega 3s), may have deleterious effects on metabolic, physical, and psychological functioning in chronic stroke survivors. However, the majority of the available literature is limited to cross-sectional studies examining the relationship between individual nutrients and recovery outcomes, which does not imply causation. Clinicians should consider the careful evaluation of dietary intake, especially among those with eating disabilities and preexisting malnutrition, in order to identity individuals at increased nutritional risk. A recent review summarizes the promising effects of nutritional modification, including oral intake retraining and alteration of dining conditions, behavior modification (i.e., reducing alcohol and fat intake and increasing fruit and vegetable intake), and supplementation on secondary stroke prevention, physical functioning, depression, and cognitive function in chronic stroke survivors [77]. Thus, the identification of suboptimal nutrient intake may allow for early intervention to maximize nutritional status, aid recovery and rehabilitation, and prevent further complications after stroke.

## Figures and Tables

**Figure 1:**
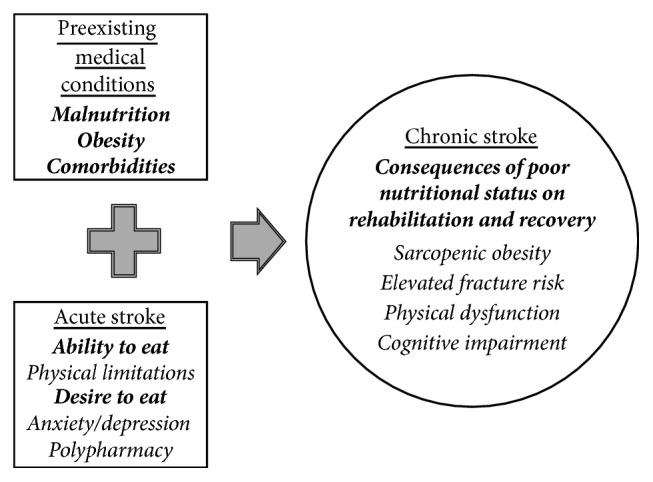
The impact of preexisting medical conditions and acute stroke consequences on dietary intake and nutritional risk during chronic stroke recovery.

**Table 1:** Nutritional considerations for the prevention of metabolic, physical, and psychological dysfunction in adult chronic stroke survivors.

Nutritional consideration	Dietary Reference Intake (United States and Canada) [25, 26]/Dietary Guidelines for Americans (United States) [27]	UL (United States and Canada) [25, 26]	Indicator of nutritional risk	Potential role in the prevention of metabolic, physical, and psychological dysfunction
*Energy and macronutrient balance*				

Energy	*Dietary guidelines* *Males*: 2,000–3,000 kcals/d, depending upon age and activity level *Females*: 1,600–2,400 kcals/d, depending upon age and activity level	ND	*Serum albumin* Reference range: 3.5–5.0 g/dL Increased risk: <3.2 g/dL *Serum prealbumin* Reference range: 15–35 mg/dL Increased risk: <15 mg/dL	(i) *Sarcopenic obesity prevention* (a) Maintenance of muscle mass (b) Prevention of excessive adipose tissue accumulation
(i) Carbohydrates	*RDA (AMDR)* *Both sexes*: 130 g/d (45–65%)	ND, but <10% of daily calories should come from added sugars		
(ii) Protein	*RDA (AMDR)* *Both sexes*: 0.8 g/kg/d (10–35%)	ND		
(iii) Fat	*AMDR* *Both sexes*: 20–35%	ND, but <10% of daily calories should come from saturated fat		

*Other nutrients*				

B-vitamins				

(i) Folate	*RDA* *Both sexes*: 400 *μ*g/d	1000 *μ*g/d	*Serum folate* Reference range: 6–20 ng/ml Deficiency: <3 ng/ml *Red blood cell folate:* Reference range: 140–628 ng/mL Deficiency: <100 ng/ml	
(ii) Vitamin B6	*RDA* *Both sexes*: 19–50 y: 1.3 mg/d *Males*: 51+ y: 1.7 mg/d *Females*: 51+ y: 1.5 mg/d	100 mg/d	*Plasma pyridoxal 5-phosphate* Reference range: 11–302 nmol/L Deficiency: <10 nmol/L	(i) *Physical dysfunction prevention* (ii) *Depressive symptom prevention* (iii) *Cognitive impairment prevention* (a) Reduced homocysteine
(iii) Vitamin B12	*RDA* *Both sexes*: 2.4 *μ*g/d^a^	ND	*Serum B12* Reference range: 500–900 pg/mL Deficiency: <200 pg/mL	
Vitamin D	*RDA* *Both sexes*: 19–70 y: 15 *μ*g/d 70+ y: 20 *μ*g/d	100 *μ*g/d	*Serum 25(OH)D* Reference range: 30–100 ng/mL Insufficiency: 20–30 ng/mL Deficiency: <20 ng/mL	(i) *Fracture prevention* (ii) *Depressive symptom prevention*
Vitamin K	*AI* *Males*: 120 *μ*g/d *Females: *90 *μ*g/d	ND	*Plasma phylloquinone* Reference range: 0.25–2.67 nmol/L Insufficiency/Deficiency: ND (<0.2 nmol/L considered “low”)	(i) *Fracture prevention*
Omega 3	*AI (α-linolenic acid)* *Males*: 1.6 g/d *Females*: 1.1 g/d	ND	*Omega 3 index ([sum of 3 omega 3 fatty acids ÷ total phospholipid fatty acids] *×* 100)* Reference range: 1.4–4.9% Increased Risk: <3.2% *Omega 6/omega 3 (sum of 6 omega 6 fatty acids ÷ sum of 3 omega 3 fatty acids)* Reference range: 5.7–21.3 Increased risk: >5	(i) *Physical dysfunction prevention* (a) Maintenance of muscle mass (b) Reduced inflammation and oxidative stress (ii) *Depressive symptom prevention* (iii) *Cognitive impairment prevention*

RDA = Recommended Dietary Allowance, AI = Adequate Intake, UL = Tolerable Upper Intake Level, AMDR = Acceptable Macronutrient Distribution Range, and ND = not determinable. ^a^Because older people may malabsorb food-bound B12, it is advisable to meet RDA through foods fortified with vitamin B12 or supplements containing B12.

## References

[1] Center for Disease Control and Prevention. Stroke Facts, http://www.cdc.gov/stroke/facts.htmAvailable from

[2] Gariballa S. E. (2000). Nutritional factors in stroke. *British Journal of Nutrition*.

[3] Dennis M. (2003). Poor nutritional status on admission predicts poor outcomes after stroke observational data from the food trial. *Stroke*.

[4] Kernan W. N., Inzucchi S. E., Sawan C., MacKo R. F., Furie K. L. (2013). Obesity: A stubbornly obvious target for stroke prevention. *Stroke*.

[5] Benzinger P., Rapp K., König H. H. (2015). Risk of osteoporotic fractures following stroke in older persons. *Osteoporosis International*.

[6] Perry L. (2004). Eating and dietary intake in communication-impaired stroke survivors: a cohort study from acute-stage hospital admission to 6 months post-stroke. *Clinical Nutrition*.

[7] Zinn S., Dudley T. K., Bosworth H. B., Hoenig H. M., Duncan P. W., Horner R. D. (2004). The effect of poststroke cognitive impairment on rehabilitation process and functional outcome. *Archives of Physical Medicine and Rehabilitation*.

[8] Benjamin E. J., Blaha M. J., Chiuve S. E., Cushman M., Das S. R., Deo R. (2017). Heart disease and stroke statistics-2017 update: a report from the american heart association. *Circulation*.

[9] Foley N. C., Salter K. L., Robertson J., Teasell R. W., Woodbury M. G. (2009). Which reported estimate of the prevalence of malnutrition after stroke is valid?. *Stroke*.

[10] Barrett A. M., Muzaffar T. (2014). Spatial cognitive rehabilitation and motor recovery after stroke. *Current Opinion in Neurology*.

[11] Jöonsson A.-C., Lindgren I., Norrving B., Lindgren A. (2008). Weight loss after stroke: A population-based study from the lund stroke register. *Stroke*.

[12] Serra M. C., Hafer-Macko C. E., Ivey F. M., Macko R. F., Ryan A. S. (2014). Impact of serum nutritional status on physical function in African American and Caucasian stroke survivors. *Stroke Research and Treatment*.

[13] Detsky A. S., McLaughlin J. R., Baker J. P. (1987). What is subjective global assessment of nutritional status?. *Journal of Parenteral and Enteral Nutrition*.

[14] Vellas B., Villars H., Abellan G., Soto ME., Rolland Y., Guigoz Y. (2006). Overview of the MNA—Its history and challenges. *The Journal of Nutrition, Health & Aging*.

[15] Peters L., O'Connor C., Giroux I., Teasell R., Foley N. (2015). Screening and assessment of nutritional status following stroke: Results from a national survey of registered dietitians in Canada. *Disability and Rehabilitation*.

[16] Bauer J. M., Vogl T., Wicklein S., Trögner J., Mühlberg W., Sieber C. C. (2005). Comparison of the mini nutritional assessment, subjective global assessment, and nutritional risk screening (NRS 2002) for nutritional screening and assessment in geriatric hospital patients. *Zeitschrift für Gerontologie und Geriatrie*.

[17] Perry L., McLaren S. (2004). An exploration of nutrition and eating disabilities in relation to quality of life at 6 months post-stroke. *Health and Social Care in the Community*.

[18] Westergren A. (2008). Nutrition and its relation to mealtime preparation, eating, fatigue and mood among stroke survivors after discharge from hospital—a pilot study. *The Open Nursing Journal*.

[19] Brynningsen P. K., Damsgaard E. M., Husted S. E. (2007). Improved nutritional status in elderly patients 6 months after stroke. *The Journal of Nutrition, Health & Aging*.

[20] Axelsson K., Asplund K., Norberg A., Eriksson S. (1989). Eating problems and nutritional status during hospital stay of patients with severe stroke. *Journal of the Academy of Nutrition and Dietetics*.

[21] Aptaker R. L., Roth E. J., Reichhardt G., Duerden M. E., Levy C. E. (Jan 1994). Serum albumin level as a predictor of geriatric stroke rehabilitation outcome. *Arch Phys Med Rehabil*.

[22] Shen H.-C., Chen H.-F., Peng L.-N. (2011). Impact of nutritional status on long-term functional outcomes of post-acute stroke patients in Taiwan. *Archives of Gerontology and Geriatrics*.

[23] Sheffler L. R., Knutson J. S., Gunzler D., Chae J. (2012). Relationship between body mass index and rehabilitation outcomes in chronic stroke. *American Journal of Physical Medicine & Rehabilitation*.

[24] Alexopoulos G. S., Meyers B. S., Young R. C., Campbell S., Silbersweig D., Charlson M. (1997). ‘Vascular depression’ hypothesis. *Archives of General Psychiatry*.

[25] (2005). *Food and Nutrition Board: Institue of Medicine. Dietary Reference Intakes for Energy, Carbohydrates, Fiber, Fat, Fatty Acids, Cholesterol, Protein, and Amino Acids*.

[26] Food and Nutrition Board IoM

[27] U.S. Department of Health and Human Services and U.S. Department of Agriculture. http://health.gov/dietaryguidelines/2015/guidelines/.

[28] Jacobsson C., Axelsson K., Wenngren B. I., Norberg A. (1996). Eating despite severe difficulties: Assessment of poststroke eating. *Journal of Clinical Nursing*.

[29] Paradiso S., Ohkubo T., Robinson R. G. (1997). Vegetative and psychological symptoms associated with depressed mood over the first two years after stroke. *International Journal of Psychiatry in Medicine*.

[30] Ostwald S. K., Wasserman J., Davis S. (2006). Medications, comorbidities, and medical complications in stroke survivors: The cares study. *Rehabilitation Nursing*.

[31] American Heart Association (2013). *Older Americans and Cardiovascular Diseases*.

[32] Ryan A. S., Dobrovolny C. L., Smith G. V., Silver K. H., Macko R. F. (2002). Hemiparetic muscle atrophy and increased intramuscular fat in stroke patients. *Archives of Physical Medicine and Rehabilitation*.

[33] De Sant’Anna M., Eboli L. C., Silva J. G. (2014). Resting metabolic rate analysis in chronic hemiparesis patients. *Neurology International*.

[34] Serra M. C., Hafer-Macko C. E. (2015). Reduced resting metabolic rate in adults with hemiparetic chronic stroke. *Journal of Neurology & Neurophysiology*.

[35] Leone A., Pencharz P. B. (2010). Resting energy expenditure in stroke patients who are dependent on tube feeding: A pilot study. *Clinical Nutrition*.

[36] Tieges Z., Mead G., Allerhand M. (2015). Sedentary behavior in the first year after stroke: A longitudinal cohort study with objective measures. *Archives of Physical Medicine and Rehabilitation*.

[37] Redfern J., McKevitt C., Dundas R., Rudd A. G., Wolfe C. D. A. (2000). Behavioral risk factor prevalence and lifestyle change after stroke: A prospective study. *Stroke*.

[38] Jang S. Y., Shin Y.-I., Kim D. Y. (2015). Effect of obesity on functional outcomes at 6 months post-stroke among elderly Koreans: A prospective multicentre study. *BMJ Open*.

[39] Doehner W., Schenkel J., Anker S. D., Springer J., Audebert H. (2013). Overweight and obesity are associated with improved survival, functional outcome, and stroke recurrence after acute stroke or transient ischaemic attack: Observations from the tempis trial. *European Heart Journal*.

[40] Vemmos K., Ntaios G., Spengos K. (2011). Association between obesity and mortality after acute first-ever stroke: The obesity-stroke paradox. *Stroke*.

[41] Donini L. M., Poggiogalle E., Migliaccio S., Pinto A., Lubrano C., Lenzi A. (2014). Sarcopenic obesity: correlation with clinical, functional, and psychological status in a rehabilitation setting. *Journal of Food and Nutrition Sciences*.

[42] Kanis J., Oden A., Johnell O. (2001). Acute and long-term increase in fracture risk after hospitalization for stroke. *Stroke*.

[43] Pouwels S., Lalmohamed A., Leufkens B. (2009). Risk of hip/femur fracture after stroke: A population-based case-control study. *Stroke*.

[44] Wu C.-H., Liou T.-H., Hsiao P.-L., Lin Y.-C., Chang K.-H. (2011). Contribution of ischemic stroke to hip fracture risk and the influence of gender difference. *Archives of Physical Medicine and Rehabilitation*.

[45] Fisher A., Srikusalanukul W., Davis M., Smith P. (2013). Poststroke hip fracture: prevalence, clinical characteristics, mineral-bone metabolism, outcomes, and gaps in prevention. *Stroke Research and Treatment*.

[46] Sato Y., Asoh T., Kondo I., Satoh K. (2001). Vitamin D deficiency and risk of hip fractures among disabled elderly stroke patients. *Stroke*.

[47] Sato Y., Iwamoto J., Honda Y. (2011). An open-label trial comparing alendronate and alphacalcidol in reducing falls and hip fractures in disabled stroke patients. *Journal of Stroke and Cerebrovascular Diseases*.

[48] Sato Y., Fujimatsu Y., Kikuyama M., Kaji M., Oizumic K. (1998). Influence of immobilization on bone mass and bone metabolism in hemiplegic elderly patients with a long-standing stroke. *Journal of the Neurological Sciences*.

[49] Yavuzer G., Ataman S., Süldür N., Atay M. (2002). Bone mineral density in patients with stroke. *International Journal of Rehabilitation Research*.

[50] Uluduz D., Adil MM., Rahim B., Gilani WI., Rahman HA., Gilani SI. (May 2014). Vitamin D deficiency and osteoporosis in stroke survivors: an analysis of National Health and Nutritional Examination Survey (NHANES). *Journal of Vascular and Interventional Neurology*.

[51] Sato Y., Tsuru T., Oizumi K., Kaji M. (1999). Vitamin K deficiency and osteopenia in disuse-affected limbs of vitamin D-deficient elderly stroke patients. *American Journal of Physical Medicine & Rehabilitation*.

[52] Pascoe M. C., Crewther S. G., Carey L. M., Noonan K., Crewther D. P., Linden T. (2012). Homocysteine as a potential biochemical marker for depression in elderly stroke survivors. *Food and Nutrition Research*.

[53] Fratoni V., Brandi M. L. (2015). B vitamins, Homocysteine and bone health. *Nutrients*.

[54] Garbagnati F., Cairella G., De Martino A. (2009). Is antioxidant and n-3 supplementation able to improve functional status in poststroke patients? Results from the nutristroke trial. *Cerebrovascular Disease*.

[55] Westergren A., Hallberg I. R., Ohlsson O. (1999). Nursing assessment of dysphagia among patients with stroke. *Scandinavian Journal of Caring Sciences*.

[56] Abbatecola A. M., Cherubini A., Guralnik J. M. (2009). Plasma polyunsaturated fatty acids and age-related physical performance decline. *Rejuvenation Research*.

[57] Reinders I., Murphy R. A., Song X. (2015). Polyunsaturated fatty acids in relation to incident mobility disability and decline in gait speed; the age, gene/environment susceptibility-reykjavik study. *European Journal of Clinical Nutrition*.

[58] Jeromson S., Gallagher I. J., Galloway S. D. R., Hamilton D. L. (2015). Omega-3 fatty acids and skeletal muscle health. *Marine Drugs*.

[59] Jensen G. L. (2008). Inflammation: roles in aging and sarcopenia. *Journal of Parenteral and Enteral Nutrition*.

[60] Millward D. J. (2012). Nutrition and sarcopenia: Evidence for an interaction. *Proceedings of the Nutrition Society*.

[61] Broomfield N. M., Quinn T. J., Abdul-Rahim A. H., Walters M. R., Evans J. J. (2014). Depression and anxiety symptoms post-stroke/TIA: prevalence and associations in cross-sectional data from a regional stroke registry. *BMC Neurology*.

[62] Hibbeln J. R. (1998). Fish consumption and major depression. *The Lancet*.

[63] Kohatsu W. (2005). Nutrition and Depression. *Explore: The Journal of Science and Healing*.

[64] Yue W., Xiang L., Zhang Y.-J., Ji Y., Li X. (2014). Association of serum 25-Hydroxyvitamin D with symptoms of depression after 6 Months in stroke patients. *Neurochemical Research*.

[65] Eyles D. W., Smith S., Kinobe R., Hewison M., McGrath J. J. (2005). Distribution of the Vitamin D receptor and 1*α*-hydroxylase in human brain. *Journal of Chemical Neuroanatomy*.

[66] Paolucci S., Antonucci G., Grasso M. G. (2001). Post-stroke depression, antidepressant treatment and rehabilitation results: a case-control study. *Cerebrovascular Disease*.

[67] Ahn D.-H., Lee Y.-J., Jeong J.-H., Kim Y.-R., Park J.-B. (2015). The effect of post-stroke depression on rehabilitation outcome and the impact of caregiver type as a factor of post-stroke depression. *Annals of Rehabilitation Medicine*.

[68] Hankey G. J., Ford A. H., Yi Q. (2013). Effect of B vitamins and lowering homocysteine on cognitive impairment in patients with previous stroke or transient ischemic attack: A prespecified secondary analysis of a randomized, placebo-controlled trial and meta-analysis. *Stroke*.

[69] Paker N., Buğdayc D., Tekdş D., Kaya B., Dere Ç. (2010). Impact of cognitive impairment on functional outcome in stroke. *Stroke Research and Treatment*.

[70] Clarke R., Smith A. D., Jobst K. A., Refsum H., Sutton L., Ueland P. M. (1998). Folate, vitamin B12, and serum total homocysteine levels in confirmed Alzheimer disease. *JAMA Neurology*.

[71] Quadri P., Fragiacomo C., Pezzati R. (2004). Homocysteine, folate, and vitamin B-12 in mild cognitive impairment, Alzheimer disease, and vascular dementia. *American Journal of Clinical Nutrition*.

[72] Rao T. S., Asha M. R., Ramesh B. N., Rao K. S. (2008). Understanding nutrition, depression and mental illnesses. *Indian Journal of Psychiatry*.

[73] Kim H., Kim G., Jang W., Kim S. Y., Chang N. (2014). Association between intake of B vitamins and cognitive function in elderly Koreans with cognitive impairment. *Nutrition Journal *.

[74] Akinyemi R. O., Allan L., Owolabi M. O. (2014). Profile and determinants of vascular cognitive impairment in African stroke survivors: the CogFAST Nigeria study. *Journal of the Neurological Sciences*.

[75] Robinson J. G., Ijioma N., Harris W. (2010). Omega-3 fatty acids and cognitive function in women. *Women's Health (London, England)*.

[76] González-Gross M., Marcos A., Pietrzik K. (2001). Nutrition and cognitive impairment in the elderly. *British Journal of Nutrition*.

[77] Serra M. C. (2017). Dietary intake and intervention in chronic stroke: review of the evidence. *Journal of Neurology & Neurophysiology*.

